# The Association between Appetitive Aggression and Social Media Addiction Mediated by Cyberbullying: The Moderating Role of Inclusive Norms

**DOI:** 10.3390/ijerph19169956

**Published:** 2022-08-12

**Authors:** Natalie Wong, Takuya Yanagida, Christiane Spiel, Daniel Graf

**Affiliations:** 1 Department of Educational Psychology, The Chinese University of Hong Kong, Hong Kong; 2 Department for Psychology of Development and Education, Faculty of Psychology, University of Vienna, Universitätsstraße 7, 1010 Vienna, Austria

**Keywords:** appetitive aggression, cyberbullying, social media addiction, inclusive norms, adolescents

## Abstract

Appetitive aggression, i.e., the motivation to obtain rewards through aggressive behaviors, has been suggested as a key driver of cyberbullying. Due to the contextual properties of cyberspace (e.g., anonymity), it is assumed that the negative effects of cyberbullying are masked, leading to a preponderance of its positive outcomes (e.g., thrill). Since cyberbullying occurs predominantly in social media, reward-learning effects may lead to problematic social media use, such as addiction. Anti-cyberbullying inclusive norms might act as a buffering factor to break this chain. However, while inclusive norms are known to reduce cyberbullying in general, their influence on the indirect effect of appetitive aggression via cyberbullying on social media addiction is yet unknown. The present study examined this indirect effect, while taking the moderating role of inclusive norms into account. A total of 1064 adolescents (42.05% male, *M*_age_ = 14.07, *SD* = 2.15) completed questionnaires. Results revealed the indirect effect of appetitive aggression on social media addiction through cyberbullying as expected. Surprisingly, this indirect effect was amplified with increasing anti-cyberbullying inclusive norms. Our findings indicate that appetitive aggression, which manifests in cyberbullying, contributes to the development of social media addiction. The unexpected results and the implications of our findings were discussed.

## 1. Introduction

As social media grows in popularity, people become increasingly reliant on social media to connect with others and to stay in touch. Although social media can be used to promote psychosocial wellbeing, such as reducing loneliness during life transitions, e.g., [1], preserving family ties [2] and coping with anxiety during COVID lockdowns, e.g., [3], excessive use and being overly reliant on social media, e.g., for stress relief or excitement, can bring around new problems. Two of such manifestations are cyberbullying perpetration and social media addiction. In this article, we examine how these two problematic behaviors may be explained by appetitive aggression, the motivation to engage in aggressive behaviors to obtain rewards [4].

Appetitive aggression has been found to be one of the most critical predictors of cyberbullying perpetration, e.g., [5,6,7]. It has been argued that appetitive aggression could be more prevalent online due to the contextual properties of cyberspace (e.g., anonymity), which masks the negative consequences of aggression, leading to a preponderance of its positive outcomes, e.g., power or thrill [4]. Since cyberbullying occurs predominantly in social media, reward-learning effects may lead to the reliance on social media for gratification, resulting in excessive and compulsive social media use. This maladaptive psychological dependence on social media is most referred to as social media addiction [8]. Although social media addiction has not been clinically classified [9], researchers generally agree that addiction-like symptoms (e.g., excessive concern about social media, losing self-control over social media use and relying on social media for stimulation) can be observed among social media users [9,10,11,12,13]. Furthermore, a number of studies have suggested that social media addiction may be classified according to the same diagnostic criteria as other addictive disorders, e.g., pathological gaming [14,15,16]. The proposed association among appetitive aggression, cyberbullying perpetration and social media addiction, if validated, could be critical in the development of effective interventions in the age of social media. 

Recent studies have demonstrated that cyberbullying perpetration and social media addiction are positively correlated [17,18,19,20,21,22]. Cyberbullying perpetration has also been studied as a predictor of social media addiction. Notably, Machimbarrena et al. (2021) found that adolescents who are involved in cyberbullying are 7.6 times more likely to display behaviors associated with social media addiction than those who are not [21]. There is also longitudinal evidence of the link between cyberbullying and social media use. Using a cross-lagged panel design, Müller et al. (2018) found that cyberbullying predicted more frequent social media use at a later time point. The association between cyberbullying and social media addiction has also been studied during the COVID pandemic [23]. A study conducted by Vejmelka and Matković (2021) found that a significant portion of adolescents (14.6%) showed moderate signs of social media addiction during the pandemic and participants who were involved in cyberbullying reported higher levels of social media addiction [22].

Despite the abundant findings on the positive association between cyberbullying perpetration and social media addiction in recent years, researchers have yet to agree upon a common motivating factor behind the two problematic behaviors. One of the major reasons behind the lack of consensus is the omission of antisocial motivation in the study of social media addiction. As shown in a recent review on social media addiction [8], studies that examine motivations that might lead to excessive and compulsive use of social media have only explored appetitive motives that are not aggressive (e.g., social gratification, self-presentation, information-seeking and entertainment). However, the prevalence of cyberbullying among youth [24] suggests that antisocial motives in social media usage could be equally important in the study of social media addiction.

We argue that appetitive aggression is a missing link that could help fill the gap in current research. We build our assumptions on the Uses and Gratification Theory (UGT) [25]. The UGT can be potentially useful in examining the proposed links and provides a useful framework to study the association between motivations and continuous social media usage. By utilizing the UGT, the present study examines the association between appetitive aggression and social media addiction, considering the mediating role of cyberbullying perpetration. As an exploratory research question and to expand our understanding of the mitigating effect of positive peer influences on cyberbullying and social media addiction, we also examined the role of inclusive norms in moderating the proposed pathways.

### 1.1. The Uses and Gratification Theory and Social Media Addiction

The Uses and Gratification Theory (UGT) was initially developed to analyse the motivations and gratifications associated with the use of different media outlets [25]. The model has since been used to examine factors underlying continuous media use and has been widely applied to study social media addiction [8]. The model has two major assumptions. First, people make reasoned choices regarding media use based on their wants and needs, e.g., [26,27]. Second, when users’ needs are met through media usage, they would seek to recreate the experience, resulting in more frequent media usage [28]. According to the model, it is important to examine whether the users’ needs are gratified during the process of social media use in order to achieve a comprehensive understanding of how motivations to use social media may lead to social media addiction.

However, existing studies that incorporate the UGT model often only examine the direct association between motivation and social media addiction, omitting the study of motive-matching cyber activities. Among recent UGT studies on social media addiction, researchers found that using social media for entertainment, e.g., to fulfill the need to pass time and have fun [28,29,30,31,32,33,34,35]; social gratification, e.g., to fulfill the need to belong [30,32,36]; and self-presentation, e.g., to fulfill the need for admiration [30,32,36], are associated with social media addiction. Although these studies provide some support that certain appetitive motives are linked with excessive and compulsive use of social media, little is known about how these motives are sustained or strengthened through engaging in rewarding cyber activities, leading to addiction. In one of the few studies that examine the role of different cyber activities in predicting social media addiction, Balakrishnan and Griffiths (2017) found that users’ level of social media addiction varies depending on the type of cyber activities they engage on YouTube [37]. Specifically, active use of YouTube (e.g., creating and commenting on videos) was shown to have a stronger association with YouTube addiction than passive use (e.g., video viewing). According to UGT, this finding implies that certain types of social media use are more gratifying (i.e., serving their needs to use social media or certain social media platforms), and thus have a greater influence on social media addiction. To combat social media addiction, it is crucial to examine behaviors that mediate the relationship between motives and addiction and develop effective behavioral interventions. 

Furthermore, existing studies focus mainly on appetitive motives that are not aggressive. As discussed earlier, the prevalence of cyberbullying among adolescents [24] suggest that antisocial motives are far from uncommon in social media use. To gain a more comprehensive understanding of the link between motives and excessive use of social media, we argue that it is important to study aggressive motives (i.e., appetitive aggression) and their respective motive-matching behaviors (i.e., cyberbullying) in social media use.

### 1.2. Appetitive Aggression and Cyberaggression

According to the Cyberaggression Typology Questionnaire (CATQ) [38], developed based on the quadripartite violence typology [39] to account for the different types of aggression in cyberspace, appetitive motives in cyberaggression can be divided into impulsive appetitive aggression (i.e., to achieve an immediate affective reward, such as fun) and controlled appetitive aggression (i.e., to achieve a desired reward). Comprehensive reviews on the role of social media affordances in online interaction, e.g., [4,40], summarized a number of features that could exacerbate these two types of appetitive aggression in online interactions. For example, while the high visualization of rewards (e.g., number of likes and views) may help to raise the sense of thrill in engaging in cyberaggression, i.e., stronger impulsive appetitive motives [40], the ease and normative ways to establish anonymity online may lead people to perceive a lower sense of consequences when they want to inflict harm on others for a desired reward such as popularity, i.e., stronger controlled appetitive motives [4]. Other unique features of social media, such as asynchronicity in communication and the paucity of social emotional cues, have also been suggested to mask the negative consequences of engaging in cyberaggression, leading to the preponderance of the rewards of engaging in cyberbullying [4,40]. As such, appetitive aggression has been suggested to be an important predictor of cyberbullying.

Accordingly, impulsive appetitive aggression has been shown to be one of the most important predictors of cyberbullying perpetration, e.g., [5,6,7,41,42]. Cyberbullying studies have shown that impulsive appetitive motives (e.g., fun-seeking tendencies about cyberaggression) are more strongly related to cyberbullying than moral beliefs in both adolescents [6] and emerging adults [7], demonstrating how important impulsive motives are in driving cyberbullying. Comparison studies on cyberbullying and face-to-face bullying also uncovered the unique role of impulse appetitive aggression in explaining aggression in cyberspace. In a recent study comparing traditional bullying and cyberbullying, impulsive appetitive motives were found to be more strongly related to cyberbullying than offline bullying [5]. Similarly, sensation-seeking tendencies were found to be more strongly associated with cyberbullying than face-to-face bullying [41]. 

There is also some evidence for the positive association between controlled appetitive aggression and cyberbullying. In a longitudinal study on the association between sociometric status and cyberbullying, Wegge et al. (2016) found that cyberbullying perpetration predicted perceived popularity at a later time point but not the other way around [43]. This finding suggests that internet users may engage in cyberbullying perpetration to acquire popularity among peers. Similarly, the need for popularity was found to be positively associated with the distribution of hurtful images of peers online [44]. Cyberbullying was also studied as one of the strategies used to gain popularity during school transition from elementary school to middle school [45]. Taken together, both impulsive and controlled appetitive aggression are shown to be significant motivating factors of cyberbullying. 

### 1.3. Present Study

Although studies on social media addiction seldom look at anti-social motivations in social media use, the significant association between appetitive motives (e.g., entertainment, social gratification and self-presentation) and social media addiction suggests that both impulsive and controlled appetitive motives are critical predictors of social media addiction. Taking the strong association among appetitive motives, cyberbullying and social media addiction into account, we hypothesize that both controlled and impulsive appetitive aggression are positively associated with cyberbullying perpetration (Hypothesis 1a and 1b) and social media addiction (Hypothesis 2a and 2b).

According to the UGT framework, experience of gratification through social media use leads to continuous usage intention. As such, cyberbullying mediates the relationship between appetitive aggression and social media addiction (Hypothesis 3). This applies to both controlled appetitive aggression (Hypothesis 3a) and impulsive appetitive aggression (Hypothesis 3b). 

#### The Moderating Role of Inclusive Norms

**Figure 1:**
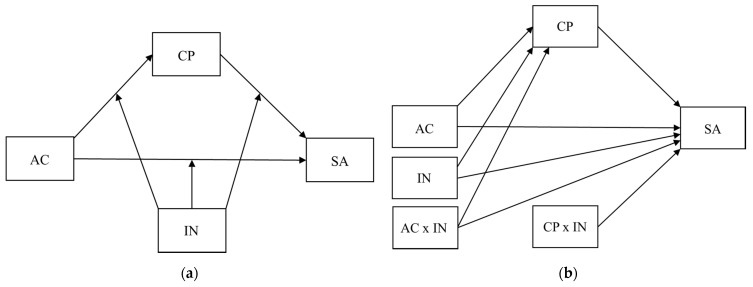
Moderated mediation model tested in the present study: (**a**) conceptual diagram; (**b**) statistical diagram. *Note*. AC = appetitive cyberaggression, CP = cyberbullying perpetration, SA = social media addiction, IN = inclusive norms.

To extend our understanding of positive peer influences in problematic social media use, we also consider the potential moderating role of inclusive norms in the pathways from appetitive aggression to social media addiction. Inclusion norms describe the degree to which people in an environment are inclusive of others. They have been shown to be critical in reducing aggressive behaviors and promoting prosocial behaviors in studies on bullying and discrimination. In a study on the belief systems of bystanders of cyberbullying, Leung, Wong and Farver (2018) found that social media users feel more obligated and confident to help cyberbullied victims when they see other people defending the victim [46]. Similarly, participants of the cyberball game (i.e., a ball-tossing game used to test the effect of social exclusion) also tend to be more inclusive of the bullied victim after seeing other peers defending them [47]. The importance of inclusive norms was also demonstrated in an earlier study on children’s acceptance towards people of other ethnicities. Specifically, Nesdale et al. (2005) found that empathy was associated with higher levels of acceptance of other ethnicities, but only when the social norms within their own group were inclusive [48]. Promoting inclusive school environments was also shown to be effective in reducing bullying behaviors against minorities [49]. However, most studies examining the moderating effect of inclusive norms do not distinguish between controlled and impulsive appetitive aggression. In view of their unique contribution to the phenomenon of cyberbullying [4], we examined the moderating effects of inclusive norms on both types of appetitive aggression and their respective associations with cyberbullying perpetration in the present study. Inclusive norms may also moderate the pathway between cyberbullying perpetration and social media addiction. As suggested in the UGT framework, a user is more motivated to engage in certain media use when it is perceived to be gratifying. As social media users feel more obliged to help the cyberbullied victim when the environment is more inclusive, e.g., [46], inclusive norms might potentially reduce the gratification of engaging in cyberbullying, leading to less addiction to social media. Figure 1 presents the moderated mediation model tested in the present study. Based on previous research on inclusive norms, inclusive norms are expected to moderate the pathway between cyberbullying perpetration and social mediation addiction (Hypothesis 4), the pathway between appetitive motives (i.e., controlled and impulsive appetitive aggression) and cyberbullying perpetration (Hypothesis 5a and 5b, respectively), and the pathway between appetitive motives and social media addiction (Hypothesis 6a and 6b, respectively). Figure 1 presents the moderated mediation model tested in the present study. 

## 2. Materials and Methods

Sample and procedure: A total of 1064 adolescents (42.05% male, *M*_age_ = 14.07, *SD* = 2.15) from 71 school classes answered questionnaires during regular school hours in the presence of a research assistant from March to November 2020. The study was conducted in accordance with the Declaration of Helsinki, and approved by the respective school boards of the federal states of lower and upper Austria. A written informed consent from the parents and the students was obtained. A total of 0.04% of data were missing, stemming from13 incomplete records. The percentage of missing values across the 31 variables ranged from 0.00 to 0.47%. 

### 2.1. Measures

#### 2.1.1. Cyberbullying Perpetration

Cyberbullying perpetration was measured with the European Cyberbullying Intervention Project Questionnaire (ECIPQ) [50]. Participants indicated whether they have ever cyberbullied others and how often they did so on a five-point Likert scale (1 = no, 2 = yes, once or twice, 3 = yes, once or twice a month, 4 = yes, about once a week, 5 = yes, more than once a week). The scale includes 11 items. A sample item is “I spread rumors about someone on the Internet”. The ECIPQ has been structurally validated in six countries [50]. The Ordinal Cronbach’s α coefficient for the cyberbullying perpetration scale was .91. 

#### 2.1.2. Appetitive Cyberaggression 

The controlled-appetitive and impulsive-appetitive cyberaggression subscales from the Cyber Aggression Typology Questionnaire (CATQ) [38,51] were used to assess participants’ level of appetitive cyberaggression in the present study. Participants rated eight items on a four-point scale (1 = not at all true of me, 2 = partly true of me, 3 = fairly true of me, 4 = very true of me). Sample items for controlled-appetitive and impulsive-appetitive cyberaggression include “Sometimes I can be mean to people online to get what I want” and “When I have fun online, I overdo it and others think I am a cyberbully or a troll”, respectively. The Ordinal Cronbach’s α coefficients for controlled-appetitive and impulsive-appetitive cyberaggression subscales were .86 and .87, respectively. 

#### 2.1.3. Inclusive Norms in Cyberspace 

Participants indicated their perceived level of inclusiveness of the cyberspace on a seven-point Likert scale (1 = not true at all; 7 = completely true). The scale has three items. A sample item is “Others would not like it if I sent mean comments to others online (using a computer or a smartphone).” The items are based on Ho et al. (2017) [52]. The alpha for our sample was .95. 

#### 2.1.4. Social Media Addiction 

Social media addiction was assessed via the Social Media Disorder Scale [53]. Participants indicated whether they suffer symptoms of social media addiction in the past year. The scale has nine dichotomous questions (i.e., yes or no). A sample item is “Did you often feel bad when you couldn’t use social media?” We used the sum score of the social media addiction scale in the moderated mediation analysis in our present study. The Ordinal Cronbach’s α of the scale was .85.

### 2.2. Measurement Models

Confirmatory factor analysis (CFA) [54] was conducted in Mplus 8.5 [55] to test the measurement models for the present study. CFA with ordered categorical indicators using robust weighted least squares estimator (WLSMV) was applied in order to take into account the ordered categorical nature of the scale items [56]. Measurement models were evaluated using the fit indices CFI, TLI, RMSEA, and SRMR based on common cut-off criteria [57]. 

The results revealed a good model fit for cyberbullying perpetration (χ^2^ (44) = 101.35, CFI = .971, TLI = .963, RMSEA = .035, and SRMR = .078), with standardized factor loadings ranging from .57 to .86. Similarly, the measurement model for appetitive cyberaggression comprising controlled-appetitive and impulsive-appetitive cyberaggression exhibited good model fit (χ^2^ (19) = 37.58, CFI = .993, TLI = .989, RMSEA = .030, and SRMR = .032), with standardized factor loadings ranging from .71 to .84 for controlled-appetitive cyberaggression and standardized factor loadings ranging from .74 to .84 for appetitive cyberaggression. Likewise, the measurement model for social media addiction exhibited good model fit (χ^2^ (27) = 75.40, CFI = .964, TLI = .952, RMSEA = .041, and SRMR = .054), with standardized factor loadings ranging from .53 to .89. In sum, the results revealed a good model fit for all scales, indicating that all scales had sound measurement properties.

### 2.3. Analytic Strategies

First, we examined the correlations between appetitive aggression and cyberbullying perpetration (Hypothesis 1a and 1b) and with social media addiction (Hypothesis 2a and 2b). Statistical mediation analysis [58] was conducted to investigate the indirect effects of appetitive cyberaggression on social media addiction via cyberbullying perpetration (Hypotheses 3a and 3b). In the second step, moderated mediation analysis [59] (Model 59) was conducted to investigate the moderating role of inclusive norms in the pathway between cyberbullying perpetration and social mediation addiction (Hypothesis 4), in the pathway between appetitive motives and cyberbullying perpetration (Hypothesis 5a and 5b), and in the pathway between appetitive motives and social media addiction (Hypothesis 6a and 6b). More specifically, interaction terms (appetitive cyberaggression x inclusive norms and cyberbullying perpetration x inclusive norms) were added to the mediation model to test for moderated mediation. All mediation models were estimated for controlled-appetitive and impulsive-appetitive cyberaggression separately. Statistical significance of the indirect effects was tested using Johnson–Neyman procedure in order to test the indirect effect for statistical significance using confidence bands along the range of the moderator variable [60].

Model parameters were estimated in Mplus 8.5 [55] using the maximum likelihood method. Statistical significance of the indirect effects was tested using bias-corrected bootstrapping confidence intervals based on 5000 bootstrap draws. The hierarchical data structure (i.e., students nested in classes) was taken into account by using cluster-robust standard errors taking into account non-independence of observations.

## 3. Results

### 3.1. The Associations of Appetitive Cyberaggression with Cyberbullying and Social Media Addiction

Correlation coefficients, means and standard deviations for all variables used in the present study are shown in Table 1. As expected, results show that cyberbullying perpetration was positively related to controlled appetitive (*r* = .66, Hypothesis 1a), impulsive appetitive cyberaggression (*r* = .60, Hypothesis 1b), and social media addiction (*r* = .28). Social media addiction was also positively related to controlled appetitive (*r* = .26, Hypothesis 2a) and impulsive appetitive cyberaggression (*r* = .23, Hypothesis 2b). These correlations are in line with our mediation hypothesis.

### 3.2. Indirect Associations between Appetitive Cyberaggression and Social Media Addiction 

Results of the mediation analysis are shown in Table 2 and Table 3. In line with our hypotheses, results of the statistical mediation analysis showed a statistically significant indirect effect of controlled-appetitive cyberaggression on social media addiction through cyberbullying perpetration (Est. = 0.94, 95% CI [0.43, 1.51], Std. Est. = 0.13, Hypothesis 3a) and a statistically significant indirect effect of impulsive appetitive cyberaggression on social media addiction through cyberbullying perpetration (Est. = 0.72, 95% CI [0.42, 1.07], Std. Est. = 0.13, Hypothesis 3b).

### 3.3. Moderating Role of Inclusive Norms on the Indirect Associations

**Figure 2:**
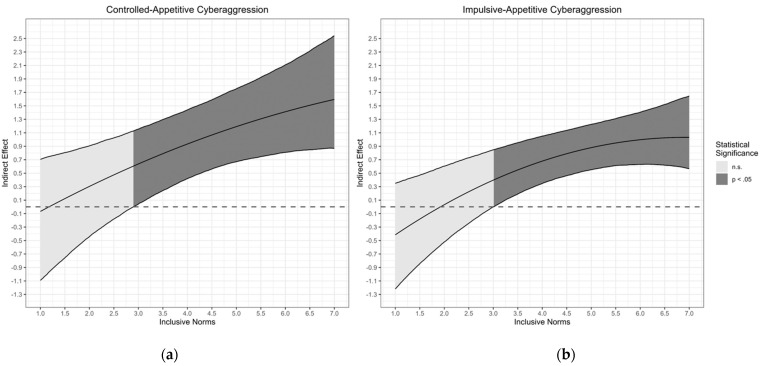
Johnson-Neyman Plot: indirect effect as a function of inclusive norms. (**a**) Controlled-appetitive cyberaggression; (**b**) impulsive-appetitive cyberaggression. *Note.* The lines represent the indirect effect conditioned on inclusive norms and lower and upper bounds of the 95% confidence bands. The dark gray areas represent the regions of statistical significance for the indirect effect at α = .05.

Results of the moderated mediation analysis for controlled-appetitive cyberaggression are shown in Table 4. In line with Hypothesis 4, results showed a statistically significant interaction between cyberbullying perpetration and inclusive norms in predicting social media addiction (Est. = 0.56, 95% CI [0.24, 0.86], Std. Est. = 0.18), indicating that the association between cyberbullying and social media addiction was stronger for students with high inclusive norms compared with students with low inclusive norms. However, there was no statistically significant interaction between controlled-appetitive cyberaggression and inclusive norms in predicting cyberbullying perpetration (Est. = −0.03, 95% CI [−0.10, 0.04], Std. Est. = −0.04, Hypothesis 5a) and no statistically significant interaction between controlled-appetitive cyberaggression and inclusive norms in predicting social media addiction (Est. = −0.12, 95% CI [−0.46, 0.16], Std. Est. = −0.04, Hypothesis 6a). The Johnson–Neyman plot depicted in Figure 1 revealed that the indirect effect becomes stronger with increasing inclusive norm, where the indirect effect is statistically significant when inclusive norms were above 2.89 (see Figure 2).

Results of the moderated mediation analysis for impulsive-appetitive cyberaggression are shown in Table 5. Again, results showed a statistically significant interaction between cyberbullying perpetration and inclusive norms in predicting social media addiction (Est. = 0.78, 95% CI [0.44, 1.10], Std. Est. = 0.25, Hypothesis 4), indicating that the association between cyberbullying and social media addiction was stronger for students with high inclusive norms compared with students with low inclusive norms. Moreover, the interaction between impulsive-appetitive cyberaggression and inclusive norms in predicting cyberbullying perpetration was statistically significant (Est. = −0.37, 95% CI [−0.63, −0.08], Std. Est. = −0.15, Hypothesis 5b), indicating that the association between impulsive-appetitive cyberaggression and cyberbullying perpetration was weaker for students with high inclusive norms compared with students with low inclusive norms. In addition, there was also a statistically significant interaction between impulsive-appetitive cyberaggression and inclusive norms in predicting social media addiction (Est. = −0.05, 95% CI [−0.10, 0.00], Std. Est. = −0.34, Hypothesis 6b), indicating that the association between impulsive-appetitive cyberaggression and social media addiction was weaker for students with high inclusive norms compared with students with low inclusive norms. The Johnson–Neyman plot depicted in Figure 1 revealed that the indirect effect becomes stronger with increasing inclusive norm, where the indirect effect is statistically significant when inclusive norms are above 3.00 (see Figure 2).

## 4. Discussion

The present study examined the mediating role of cyberbullying perpetration in the association between appetitive aggression and social media addiction using The Uses and Gratification Theory (UGT). Our study filled two significant gaps in the use of UGT in social media addiction research. First, we examined antisocial appetitive motives (i.e., appetitive aggression) as a predictor of social media addiction. Second, we examined the mediating role of motive-matching behaviors (i.e., cyberbullying perpetration) in the relationship between motives and social media addiction. 

Using the UGT framework, we demonstrated that appetitive aggression could explain social media addiction through cyberbullying perpetration (Hypotheses 1–3). The finding highlights the importance of considering antisocial motives when studying social media addiction. While existing UGT studies on social media addiction identified impulsive and controlled appetitive motives that could potentially lead to social media addiction, e.g., using social media for entertainment and social gratification [8], these studies focus largely on appetitive motives that are not aggressive or antisocial. As a result of this gap in research, it has been difficult to explain the consistent and positive association between cyberbullying perpetration and social media addiction, as is often identified in recent studies, e.g., [17,18,19,20,21,22]. Utilizing the UGT framework, our study offers some support for the argument that appetitive motives to engage in cyberaggression contribute to social media addiction through actual engagement in cyberbullying perpetration. As discussed earlier, the negative consequences of cyberbullying are often masked due to unique properties of online communication, e.g., the paucity of socioemotional cues and asynchronicity in communication [4,40]. As a result, actual engagement in cyberbullying could be exceptionally gratifying and therefore very effective in motivating more social media use. The results suggest that future research and interventions on social media addiction could include appetitive aggression and cyberbullying in addition to the current focus on nonaggressive motives in social media use.

In contrast to what we expected, inclusive norms were found to enhance the association between cyberbullying and social media addiction (Hypothesis 4). While we hypothesized that inclusive norms may take away the gratification of cyberbullying others, thereby buffering the pathway between cyberbullying perpetration and social media addiction, our results suggest otherwise. This counterintuitive result could be potentially explained by cyberbullies’ expectation of being judged negatively when inclusive norms are high. Knowing that other people disapprove of cyberbullying (i.e., higher levels of inclusive norms) may lead to stronger expectation of negative judgment after engaging in cyberbullying behaviors, resulting in more addiction-like symptoms. This includes rumination about social media (e.g., ruminating about their cyberbullying behaviors), losing control over their social media use (e.g., wanting to check on other people’s feedback to their cyberbullying behaviors) as well as emotional outbursts due to expected negative judgment on their social media use (e.g., arguing with parents, brothers and sisters over social media use). The experience of such negative emotions may increase the likelihood of social media addiction being reported. While there is little research on how inclusive norms may intensify rumination about one’s own antisocial behaviors on social media, there have been studies that showed that feeling inferior to others (i.e., negative social comparison) may lead to more social media rumination, which results in higher levels of compulsive social media use, e.g., [61]. Although the expectations of being judged negatively might explain the aggravating effect of inclusive norms of the association between cyberbullying perpetration and social media addiction, more research is needed to examine this hypothesis. The paradoxical role played by inclusive norms should be further explored in future research.

To expand our understanding of positive peer influences in problematic social media use, we also explored the moderating role of inclusive norms in the pathways between appetitive aggression and social media addiction. Accordingly, we found partial support for the moderating role of inclusive norms on the associations between appetitive aggression and (1) cyberbullying perpetration (Hypothesis 5) and (2) social media addiction (Hypothesis 6). Specifically, it was found that inclusive norms can buffer the associations between impulsive appetitive aggression and cyberbullying as well as social media addiction. However, the interaction between controlled appetitive aggression and inclusive norms was not significant in explaining cyberbullying perpetration nor social media addiction. Based on the UGT framework, the discrepancy found between impulsive appetitive aggression and controlled appetitive aggression might be explained by whether inclusive norms can buffer the gratifying effect of engaging in cyberaggression. Our findings suggest that even though higher perceived inclusive norms may dampen the immediate affective rewards for cyberbullying others (i.e., taking away the fun of cyberaggression), this dampening effect may be weaker on other desired rewards, such as getting attention and asserting dominance over others. Relatedly, a recent study on bullying among adolescents found that while bullies have higher popularity goals and social status insecurity, their social preference goal (i.e., the need to be liked by peers) is not significantly higher [62]. This finding may explain why controlled appetitive aggression, such as the motive to assert dominance using cyberaggression, may outweigh their perceived repercussions of being disliked by their peers for excluding others. 

This study has a few limitations. First, our (moderated) mediation analyses are conducted using cross-sectional data. While the tested model is based on established theories (e.g., UGT) and results from previous studies, our findings should still be interpreted with caution as (1) the sequence of relationships is assumed rather than tested using a longitudinal design and (2) it is unclear whether the sequence of relationships represents causal effects, i.e., mediation involves causal processes that occur over time [63,64]. Future studies with longitudinal (e.g., cross-lagged models) or experimental designs could be helpful in verifying our findings. Second, the scale used to measure inclusive norms does not distinguish between opinions of people with different relationships with the participant. Participants were only asked to report the general attitudes of others towards cyberbullying and exclusion. This could be an issue as recent studies have shown that others’ opinions on social media use may only be influential when the opinions are from people close to them, e.g., close friends and family [65]. Future studies should compare the opinions of close and distant others in order to test our findings on inclusive norms. Third, we did not account for personality traits that may also contribute to the link between appetitive aggression and social media addiction. Several studies that examine the association between personality traits and social media addiction reported that agreeableness was negatively associated with excessive and compulsive social media use, e.g., [28,31,66,67,68]. These findings suggest that low levels of agreeableness may be a risk factor of social media addiction. As such, future studies on personality factors are needed to deepen our understanding of individual differences in the link between appetitive aggression and social media addiction.

## 5. Conclusions

Our study has demonstrated the usefulness of UGT in explaining the pathways from appetitive aggression to cyberbullying perpetration to social media addiction. Accordingly, we showed that appetitive aggression can lead to more social media addiction through the engagement of gratifying activities (i.e., cyberbullying perpetration). The results of our study suggest that addressing appetitive aggression in future interventions could be helpful in reducing both cyberbullying perpetration and social media addiction. In terms of the role of positive peer environment, we found that inclusive norms may buffer the relationship between appetitive aggression and problematic behaviors on social media via a stronger perception of prosocial inclusive norms. This attenuation could be due to a reduction in the gratification experienced through cyberbullying. However, inclusive norms may intensify the association between cyberbullying preparation and social media addiction due to the expectation of negative judgements from others. The paradoxical effect of inclusive norms on the association between cyberbullying and social media addiction identified in this study highlights the importance of further research on peer influence in problematic social media use.

## Figures and Tables

**Table 1:** Descriptive Statistics: Bivariate correlations, Means, and Standard Deviations.

Scale	M	SD	Correlation Coefficients				
			1.	2.	3.	4.	5.
1. Cyberbullying perpetration	1.12	0.24					
2. Controlled appetitive cyberaggression	1.10	0.27	**.66**				
3. Impulsive appetitive cyberaggression	1.17	0.37	**.60**	**.63**			
4. Inclusive norms	5.57	2.01	**−.16**	**−.21**	**−.17**		
5. Social media addiction	2.06	2.00	**.28**	**.26**	**.23**	−.03	

*Note. N* = 1064. Statistically significant results at α = .05 are in boldface.

**Table 2:** Results of the Mediation Analysis for Controlled-Appetitive Cyberaggression: Indirect and Direct Effect.

Independent Variable	Mediating Variable	Dependent Variable	Est. (SE)	95% CI	Std. Est.
*Individual components of the indirect and direct effects*					
Controlled-AC→	CP		**0.59** (0.07)	[0.47, 0.72]	0.66
	CP	→SA	**1.60** (0.54)	[0.65, 2.77]	0.19
*Indirect effects*					
Controlled-AC→	CP	→SA	**0.94** (0.28)	[0.43, 1.51]	0.13
*Direct effect controlling for the indirect effect*					
Controlled-AC		→SA	**0.98** (0.34)	[0.34, 1.69]	0.13

*Note.* Est. = unstandardized parameter estimate; SE = standard error; 95% CI = 95% bias-corrected bootstrap confidence interval; Std. Est. = standardized estimate; Controlled-AC = controlled-appetitive cyberaggression; CP = cyberbullying perpetration; SA = social media addiction; statistically significant results at α = .05 are in boldface; → indicates the direction of the pathway between two variables; R^2^_CP_ = .43; R^2^_SA_ = .09.

**Table 3:** Results of the Mediation Analysis for Impulsive-Appetitive Cyberaggression: Indirect and Direct Effect.

Independent Variable	Mediating Variable	Dependent Variable	Est. (SE)	95% CI	Std. Est.
*Individual components of the indirect and direct effects*					
Impulsive-AC→	CP		**0.40** (0.05)	[0.30, 0.48]	0.60
	CP	→SA	**1.83** (0.48)	[0.98, 2.88]	0.22
*Indirect effects*					
Impulsive-AC→	CP	→SA	**0.72** (0.17)	[0.42, 1.07]	0.13
*Direct effect controlling for the indirect effect*					
Impulsive-AC		→SA	**0.54** (0.27)	[0.02, 1.08]	0.10

*Note.* Est. = unstandardized parameter estimate; SE = standard error; 95% CI = 95% bias-corrected bootstrap confidence interval; Std. Est. = standardized estimate; impulsive-AC = impulsive-appetitive cyberaggression; CP = cyberbullying perpetration; SA = social media addiction; statistically significant results at α = .05 are in boldface; → indicates the direction of the pathway between two variables; R^2^_CP_ = .36; R^2^_SA_ = .09.

**Table 4:** Results of the Moderated Mediation Analysis for Controlled-Appetitive Cyberaggression (Controlled-AC): Indirect and Direct Effects Moderated by Inclusive Norms (IN).

Independent Variable.	Mediating Variable	Dependent Variable	Est. (SE)	95% CI	Std. Est.
*Individual components of the indirect and direct effects*					
Controlled-AC→	CP		**0.54** (0.05)	[0.45, 0.65]	0.30
IN→	CP		0.00 (0.00)	[−0.01, 0.00]	0.04
Controlled-AC × IN→	CP		−0.03 (0.04)	[−0.10, 0.04]	−0.04
	CP	→SA	**2.47** (0.54)	[1.44, 3.57]	0.30
IN		→SA	0.04 (0.03)	[−0.03, 0.10]	0.04
	CP × IN	→SA	**0.56** (0.15)	[0.24, 0.86]	0.18
Controlled-AC × IN		→SA	−0.12 (0.16)	[−0.46, 0.16]	−0.04
*Indirect effects given average level of* *inclusive norms (i.e., M = 5.57)*					
Controlled-AC→	CP	→SA	**1.32** (0.30)	[0.76, 1.95]	0.08
*Direct effect controlling for the indirect effect given average level of inclusive norm (i.e., M = 5.57)*					
Controlled-AC		→SA	**0.89** (0.37)	[0.19, 1.67]	0.12

*Note.* Est. = unstandardized parameter estimate; SE = standard error; 95% CI = 95% bias-corrected bootstrap confidence interval; Std. Est. = standardized estimate; Controlled-AC = controlled-appetitive cyberaggression; IN = inclusive norms; CP = cyberbullying perpetration; SA = social media addiction; statistically significant results at α = .05 are in boldface; → indicates the direction of the pathway between two variables; R^2^_CP_ = .44; R^2^_SA_= .11.

**Table 5:** Results of the Moderated Mediation Analysis for Impulsive-Appetitive Cyberaggression (Impulsive-AC): Indirect and Direct Effects Moderated by Inclusive Norms (IN).

Independent Variable.	Mediating Variable	Dependent Variable	Est. (SE)	95% CI	Std. Est.
*Individual components of the indirect and direct effects*					
Impulsive-AC→	CP		**0.34** (0.04)	[0.27, 0.41]	0.51
IN→	CP		**−0.01** (0.00)	[−0.02, −0.00]	−0.18
Impulsive-AC × IN→	CP		**−0.05** (0.03)	[−0.10, 0.00]	−0.34
	CP	→SA	**2.85** (0.49)	[1.87, 3.83]	0.35
IN		→SA	0.03 (0.03)	[−0.04, 0.10]	0.03
	CP × IN	→SA	**0.78** (0.17)	[0.44, 1.10]	0.25
Impulsive-AC × IN		→SA	**−0.37** (0.14)	[−0.63, −0.08]	−0.15
*Indirect effects given average level of* *inclusive norm (i.e., M = 5.57)*					
Impulsive-AC→	CP	→SA	**0.96** (0.18)	[0.62, 1.33]	0.18
*Direct effect controlling for the indirect effect given average level of inclusive norm (i.e., M = 5.57)*					
Impulsive-AC		→SA	0.27 (0.27)	[−0.23, 0.82]	0.05

*Note.* Est. = unstandardized parameter estimate; SE = standard error; 95% CI = 95% bias-corrected bootstrap confidence interval; Std. Est. = standardized estimate; Impulsive-AC = impulsive appetitive cyberaggression; IN = inclusive norms; CP = cyberbullying perpetration; SA = social media addiction; statistically significant results at α = .05 are in boldface; → indicates the direction of the pathway between two variables; R^2^ _CP_ = .39; R^2^_SA_ = .11.

## Data Availability

The data presented in this study are available on request from the corresponding author.
